# *MYCN* amplification drives an aggressive form of spinal ependymoma

**DOI:** 10.1007/s00401-019-02056-2

**Published:** 2019-08-14

**Authors:** David R. Ghasemi, Martin Sill, Konstantin Okonechnikov, Andrey Korshunov, Stephen Yip, Peter W. Schutz, David Scheie, Anders Kruse, Patrick N. Harter, Marina Kastelan, Marlies Wagner, Christian Hartmann, Julia Benzel, Kendra K. Maass, Mustafa Khasraw, Ronald Sträter, Christian Thomas, Werner Paulus, Christian P. Kratz, Hendrik Witt, Daisuke Kawauchi, Christel Herold-Mende, Felix Sahm, Sebastian Brandner, Marcel Kool, David T. W. Jones, Andreas von Deimling, Stefan M. Pfister, David E. Reuss, Kristian W. Pajtler

**Affiliations:** 1 Hopp-Children’s Cancer Center Heidelberg (KiTZ), Heidelberg, Germany; 2 Division of Pediatric Neurooncology, German Cancer Research Center (DKFZ), German Cancer Consortium (DKTK), Heidelberg, Germany; 3 Clinical Cooperation Unit Neuropathology, German Cancer Research Center (DKFZ), German Consortium for Translational Cancer Research (DKTK), Heidelberg, Germany; 4 Department of Neuropathology, Institute of Pathology, Heidelberg University Hospital, Heidelberg, Germany; 5 Pathology and Laboratory Medicine, University of British Columbia, Vancouver, BC Canada; 6 Department of Pathology, Rigshospitalet, Copenhagen, Denmark; 7 Spine Section, Department of Orthopedic Surgery, Rigshospitalet, Copenhagen, Denmark; 8 Institute of Neurology (Edinger-Institute), University Hospital Frankfurt, Goethe University, Frankfurt am Main, Germany; 9 German Cancer Consortium (DKTK), Partner Site Frankfurt/Mainz, German Cancer Research Center (DKFZ), Heidelberg, Germany; 10 Frankfurt Cancer Institute (FCI), Frankfurt am Main, Germany; 11 Northern Sydney Cancer Centre, Royal North Shore Hospital, Sydney, NSW Australia; 12 The Brain Cancer Group, Sydney, NSW Australia; 13 LOEWE Center for Personalized Translational Epilepsy Research (CePTER), Frankfurt, Germany; 14 Institute of Neuroradiology, Goethe University Hospital Frankfurt, Frankfurt, Germany; 15 Department of Neuropathology, Hannover Medical School, Hannover, Germany; 16 Department of Pediatric Oncology, Hematology, and Immunology, University Hospital Heidelberg, Heidelberg, Germany; 17 Royal North Shore Hospital, The University of Sydney, Sydney, Australia; 18 Department of Pediatric Hematology/Oncology, University of Münster, Münster, Germany; 19 Institute of Neuropathology, University Hospital Münster, Münster, Germany; 20 Department of Pediatric Hematology and Oncology, Hannover Medical School, Hannover, Germany; 21 Department of Neurosurgery, Heidelberg University Hospital, Heidelberg, Germany; 22 Division of Neuropathology, National Hospital for Neurology and Neurosurgery, University College London Hospitals NHS Foundation Trust, London, UK; 23 Department of Neurodegenerative Disease, UCL Queen Square Institute of Neurology, Queen Square, London, UK; 24 Pediatric Glioma Research Group, German Cancer Research Center (DKFZ), Heidelberg, Germany

**Keywords:** Ependymoma, Intradural extramedullary ependymoma, MYCN, Spinal tumor, DNA methylation, CNS malignancies

## Abstract

Spinal ependymal tumors form a histologically and molecularly heterogeneous group of tumors with generally good prognosis. However, their treatment can be challenging if infiltration of the spinal cord or dissemination throughout the central nervous system (CNS) occurs and, in these cases, clinical outcome remains poor. Here, we describe a new and relatively rare subgroup of spinal ependymal tumors identified using DNA methylation profiling that is distinct from other molecular subgroups of ependymoma. Copy number variation plots derived from DNA methylation arrays showed *MYCN* amplification as a characteristic genetic alteration in all cases of our cohort (*n* = 13), which was subsequently validated using fluorescence in situ hybridization. The histological diagnosis was anaplastic ependymoma (WHO Grade III) in ten cases and classic ependymoma (WHO Grade II) in three cases. Histological re-evaluation in five primary tumors and seven relapses showed characteristic histological features of ependymoma, namely pseudorosettes, GFAP- and EMA positivity. Electron microscopy revealed cilia, complex intercellular junctions and intermediate filaments in a representative sample. Taking these findings into account, we suggest to designate this molecular subgroup spinal ependymoma with MYCN amplification, SP-EPN-MYCN. SP-EPN-MYCN tumors showed distinct growth patterns with intradural, extramedullary localization mostly within the thoracic and cervical spine, diffuse leptomeningeal spread throughout the whole CNS and infiltrative invasion of the spinal cord. Dissemination was observed in 100% of cases. Despite high-intensity treatment, SP-EPN-MYCN showed significantly worse median progression free survival (PFS) (17 months) and median overall survival (OS) (87 months) than all other previously described molecular spinal ependymoma subgroups. OS and PFS were similar to supratentorial ependymoma with RELA-fusion (ST-EPN-RELA) and posterior fossa ependymoma A (PF-EPN-A), further highlighting the aggressiveness of this distinct new subgroup. We, therefore, propose to establish SP-EPN-MYCN as a new molecular subgroup in ependymoma and advocate for testing newly diagnosed spinal ependymal tumors for *MYCN* amplification.

**Electronic supplementary material:**

The online version of this article (10.1007/s00401-019-02056-2) contains supplementary material, which is available to authorized users.

## Introduction

Ependymoma comprises a heterogeneous group of primary central nervous system (CNS) tumors in children and adults. Based on DNA methylation profiling, ependymomas were classified into nine distinct molecular subgroups, with three in each anatomic compartment of the CNS (supratentorial, posterior fossa, and spine) [30, 31]. Spinal ependymal tumors account for 21.5% and 18.3% of all primary spinal tumors in pediatric and adult patients, respectively, as well as 3–6% of all CNS malignancies in general [9, 18, 29]. The three previously established molecular subgroups, spinal subependymoma (SP-SE), spinal myxopapillary ependymoma (SP-MPE), and spinal ependymoma (SP-EPN), show relatively good concordance with the histopathological subtypes subependymoma, myxopapillary ependymoma, and (classic) ependymoma, respectively [31]. SP-SE and SP-EPN generally arise intramedullary, while SP-MPE occur extramedullary and are almost exclusively located at the filum terminale or the conus medullaris [1, 7, 20, 37, 38]. Spinal ependymal tumors usually grow slowly and are often well demarcated. Clinical outcome is generally better than that of intracranial ependymomas, with a 5-year OS ranging from 60 to 90% [5, 18, 31]. Poor outcome has been described in some series, especially for WHO Grade III ependymoma [18, 29, 44] which tend to infiltrate the spinal cord and show aggressive biological behavior. Treatment of individuals with these tumors remains challenging due to the difficulty of achieving gross total resections, the paucity of established treatment protocols and the uncertainty regarding the therapeutic value of radiotherapy [1, 27, 44, 45]. Although spinal ependymal tumors are characterized by distinct somatic copy number variations (CNV), e.g. loss of chromosome 6q in SP-SE, 22q in SP-EPN, and general chromosomal instability in SP-MPE, recurrent oncogenic drivers especially in aggressive tumors have not yet been identified. [25, 31, 45]. Herein, we describe a novel molecular subgroup of spinal ependymal tumors using genome-wide DNA methylation analysis. These tumors invariably exhibited an aggressive clinical course and were molecularly characterized by focal high-level amplification of *MYCN*.

## Materials and methods

### Tumor material and clinical data

Tumor tissue and retrospectively collected clinical data from 13 patients with the local diagnosis of spinal ependymal tumors (made between 2003 and 2018) were obtained from multiple international collaborating centers and collected at the Department of Neuropathology of the University Hospital Heidelberg (Heidelberg, Germany). For all cases, a genotype check was performed to exclude the possibility that material from the same patient was received from more than one center. To this end, the Pearson correlation across beta methylation values of 59 rs-loci present on both the Illumina Infinium HumanMethylation450 and the Illumina Infinium HumanMethylation EPIC array were calculated. Samples with a correlation ≥ 0.95 were considered as genotype match.

Written consent by all patients or their legal representative was obtained. Research use of tissues, clinical and radiological data were in accordance with local ethical approvals.

### DNA methylation-based clustering and copy number variation plots (CNVs)

Genome-wide DNA methylation profiling was performed using the Illumina Infinium HumanMethylation450 and the Illumina Infinium HumanMethylation EPIC Kits as previously described and according to the manufacturer’s instructions [31].

All computational analyses were performed in R version 3.4.4 (R Development Core Team, 2019). Raw signal intensities were obtained from IDAT files using the minfi Bioconductor package version 1.24.0 [2, 15]. Illumina EPIC and 450k samples were merged to a combined data set by selecting the intersection of probes present on both arrays (combineArrays function, minfi). Each sample was individually normalized by performing a background correction (shifting of the 5th percentile of negative control probe intensities to 0) and a dye-bias correction (scaling of the mean of normalization control probe intensities to 10,000) for both color channels. Subsequently, a correction for the type of material tissue (FFPE/frozen) and array (450k/EPIC) was performed by fitting univariate, linear models to the log2-transformed intensity values (removeBatchEffect function, limma package version 3.34.5). The methylated and unmethylated signals were corrected individually. Beta-values were calculated from the retransformed intensities using an offset of 100 (as recommended by Illumina).

Before further analysis, the following filtering criteria were applied: removal of probes targeting the X and Y chromosomes (*n* = 11,551), removal of probes containing a single-nucleotide polymorphism (dbSNP132 Common) within five base pairs of and including the targeted CpG-site (*n* = 7998), probes not mapping uniquely to the human reference genome (hg19) allowing for one mismatch (*n* = 3965), and 450k array probes not included on the EPIC array. In total, 428,230 probes were kept for downstream analysis.

To perform unsupervised non-linear dimension reduction, the remaining probes were used to calculate the 1-variance weighted Pearson correlation between samples. The resulting distance matrix was used as input for t-Distributed Stochastic Neighbor Embedding analysis (t-SNE; Rtsne package version 0.13). The following non-default parameters were applied: theta = 0, pca = F, max_iter = 2500 perplexity = 20.

To identify fitting samples for this study, DNA-methylation profiles of 53,468 samples from different tumor entities and experimental data were screened and compared with the reference cohort of the Heidelberg brain tumor methylation classifier which is based on 2682 CNS tumors representing 82 distinct tumor methylation classes (https://www.molecularneuropathology.org) [6].

CNV analysis from 450k and EPIC methylation array data was performed using the conumee Bioconductor package version 1.12.0 (Hovestadt V, Zapatka M, 2017).

### Pathology, histology, electron microscopy, and immunohistochemistry

Hematoxylin–eosin (H&E)-stained slides were evaluated applying the diagnostic criteria provided by the 2016 WHO Classification of Tumors of the Central Nervous System [24]. Tumors were assessed histologically for the following features: cellularity, perivascular pseudorosettes, microvascular proliferation, necrosis, and mitotic activity.

Immunohistochemistry and fluorescence in situ hybridization were conducted on 1-μm thick formalin-fixed, paraffin-embedded (FFPE) tissue sections mounted on StarFrost Advanced Adhesive slides (Engelbrecht, Kassel, Germany) followed by drying at 80 °C for 15 min. Immunohistochemistry was performed on a BenchMark Ultra immunostainer (Ventana Medical Systems, Tucson, AZ, USA).

**Table:** 

	Antibody	Dilution	Pretreatment	Signal detection
GFAP	Z0334, DAKO	1:1000	None	Ultraview
EMA	GP1.4, NeoMarkers	1:1000	95 °C, CC1, 52 min.	Ultraview
Ki67	MIB-1, DAKO	1:100	92 °C, CC1, 64 min.	Optiview
H3K27me3	07–449, Millipore, Billercia, MA	1:1000	95 °C, CC1, 92 min.	Ultraview
MYCN	D4B2Y, Cell signaling	1:100	100 °C, CC1, 64 min.	Optiview

For Ki67 analysis, tumor areas with the highest Ki67 labelling indices were evaluated for the fraction of positive cell nuclei by counting all cells excluding lymphocytes and vascular cells in one 200 × microscopic field.

Tissue for ultrastructural examination in one patient was retrieved from a paraffin block, rehydrated, post-fixed in 2.5% glutaraldehyde, and processed and stained for electron microscopy as per routine protocol. Thin sections were examined on a Zeiss 910 electron-microscope.

### Fluorescence in situ hybridization (FISH)

Two-color interphase FISH was performed using a target probe for *MYCN* (2p24; green) and *AFF3* probe (red) as a reference (Zytovision SPEC *MYCN*/2q11 Dual Color Probe). Samples showing sufficient FISH efficiency (> 90% nuclei with signals) were evaluated. Signals were scored in at least 200 non-overlapping, intact nuclei. Specimens were considered amplified for *MYCN* locus when more than 10% of tumor cells exhibited either more than eight signals of the corresponding probe with a reference/control ratio > 4.0 or innumerable tight clusters of signals of the reference locus probe.

### RNA sequencing and gene expression profiling

Gene expression profiling was analyzed using two different approaches: RNA sequencing and Affymetrix arrays. RNA sequencing from fresh frozen tumor material of two patients was performed by the High Throughput Sequencing Unit of the Genomics & Proteomics Core Facility at the DKFZ using the Illumina HiSeq 2000 platform (V4: 125 bp paired end reads) and the TruSeq Stranded mRNA Library Prep Kit according to the manufacturer’s instructions. The general processing of RNA-sequencing data (reads alignment, quality control, and counts computation) was performed as previously described [26]. Gene expression profiling on the Affymetrix GeneChip U133 Plus 2.0 array was undertaken for one patient as previously described [43].

### Statistics

Age distribution and median age were calculated using R (R Core Team, 2017). Survival analysis was performed applying the Kaplan–Meier method using GraphPadPrism for Windows (Graphpad Software 8.0.2, La Jolla California USA, www.graphpad.com). *P* values comparing the survival rates of the respective molecular subgroups were calculated using log-rank tests and were rounded to three decimal digits. PFS was defined as the time interval in years between first diagnosis and progression of a local tumor or detection of distant seeding or local recurrence. OS was defined as the time interval between first diagnosis and death. Patients were censored at the point of death or loss of follow-up. For patient 13, no detailed data regarding OS and PFS were available.

## Results

### DNA methylation profiling reveals an epigenetically distinct group of spinal ependymal tumors

**Fig. 1:**
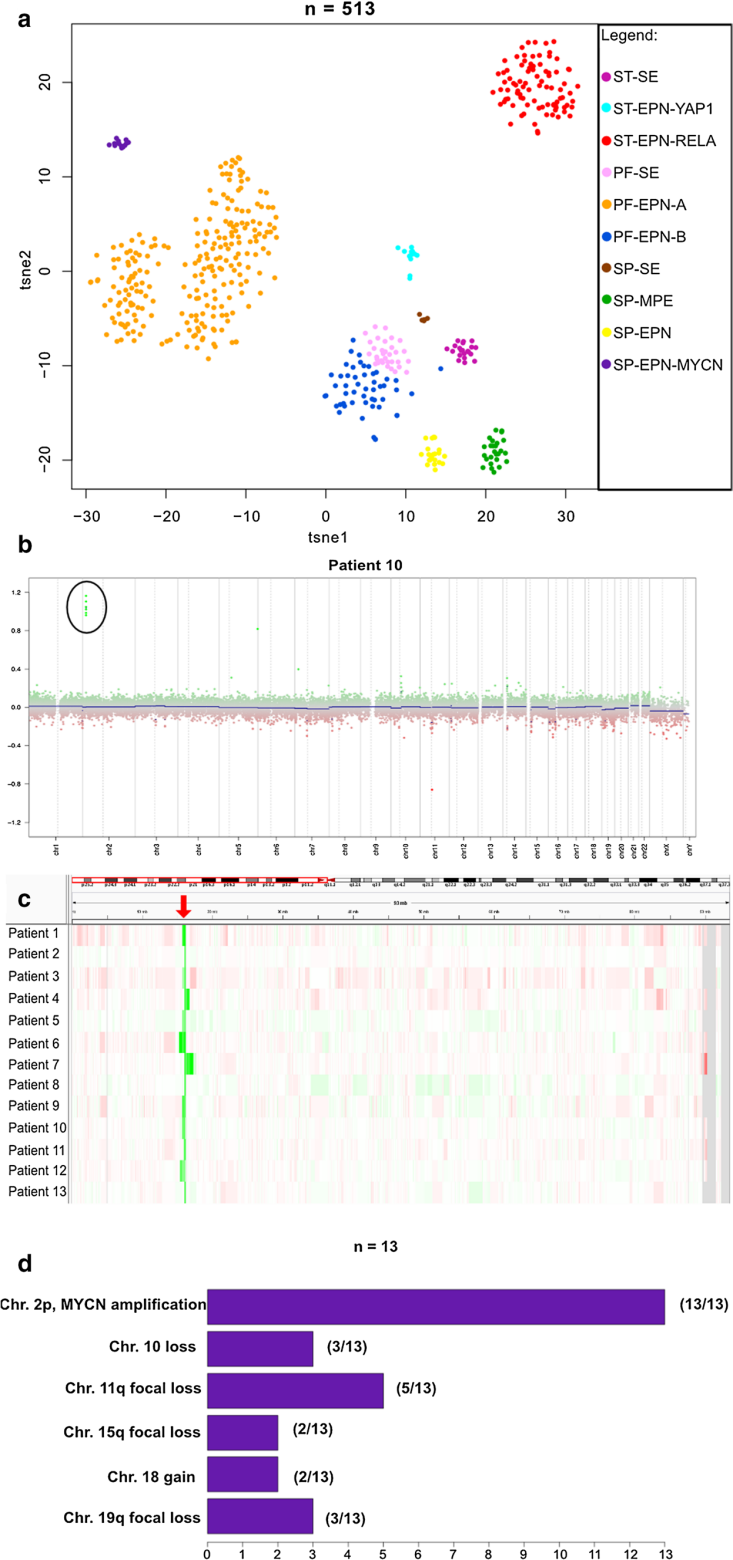
DNA-methylation based clustering and CNV analysis in SP-EPN-MYCN **a** t-SNE analysis showing DNA-methylation clustering of the SP-EPN-MYCN-cohort (*n* = 13) with 500 ependymomas of all nine major molecular subgroups. SP-EPN-MYCN (purple) shows distinct methylation patterns compared with other molecular ependymal subgroups. (Used data set for the reference cohort: Pajtler et al, Cancer Cell, 2015). **b** CNV-plot of a representative case (patient 10) showing strong *MYCN* amplification on chromosome 2p. Black circles mark amplification. **c** IGV-representation of CNV-profiling of chromosome 2p for all 13 cases showing *MYCN* amplification detected by DNA methylation profiling (red arrow). Green and red colors mark amplified and deleted regions of the genome, respectively. **d** Bar plot summarizing the most frequent CNAs detected in SP-EPN-MYCN-cases

Using a screening approach based on unsupervised analysis of DNA methylation profiling data of a large set of CNS tumors, we identified a distinct cohort of thirteen tumors histopathologically diagnosed as ependymoma. When these samples were clustered with an extensive set of 53,455 DNA methylation profiles covering more than 80 molecularly defined classes of CNS tumors, malignancies outside the CNS, and experimental data (i.e. cell lines, mouse models and patient-derived xenograft models) in a t-SNE-analysis, tumors from this cohort formed a distinct and stable cluster (data not shown) [6]. The highest predicted molecular class was posterior fossa ependymoma type A (PFA) (12/13) or posterior fossa ependymoma type B (PFB) (1/13); however, due to low calibrated scores samples were previously returned as “no matching methylation class” (calibrated score < 0.9, MNP Classifier v11b4). Next, we compared the methylation patterns of our cohort with a reference set of 500 ependymomas from all nine major molecular subgroups [31] (Fig. 1a). The new group did not cluster with any of the other previously described ependymoma subgroups.

### *MYCN* amplification is a characteristic feature of the group

**Fig. 2:**
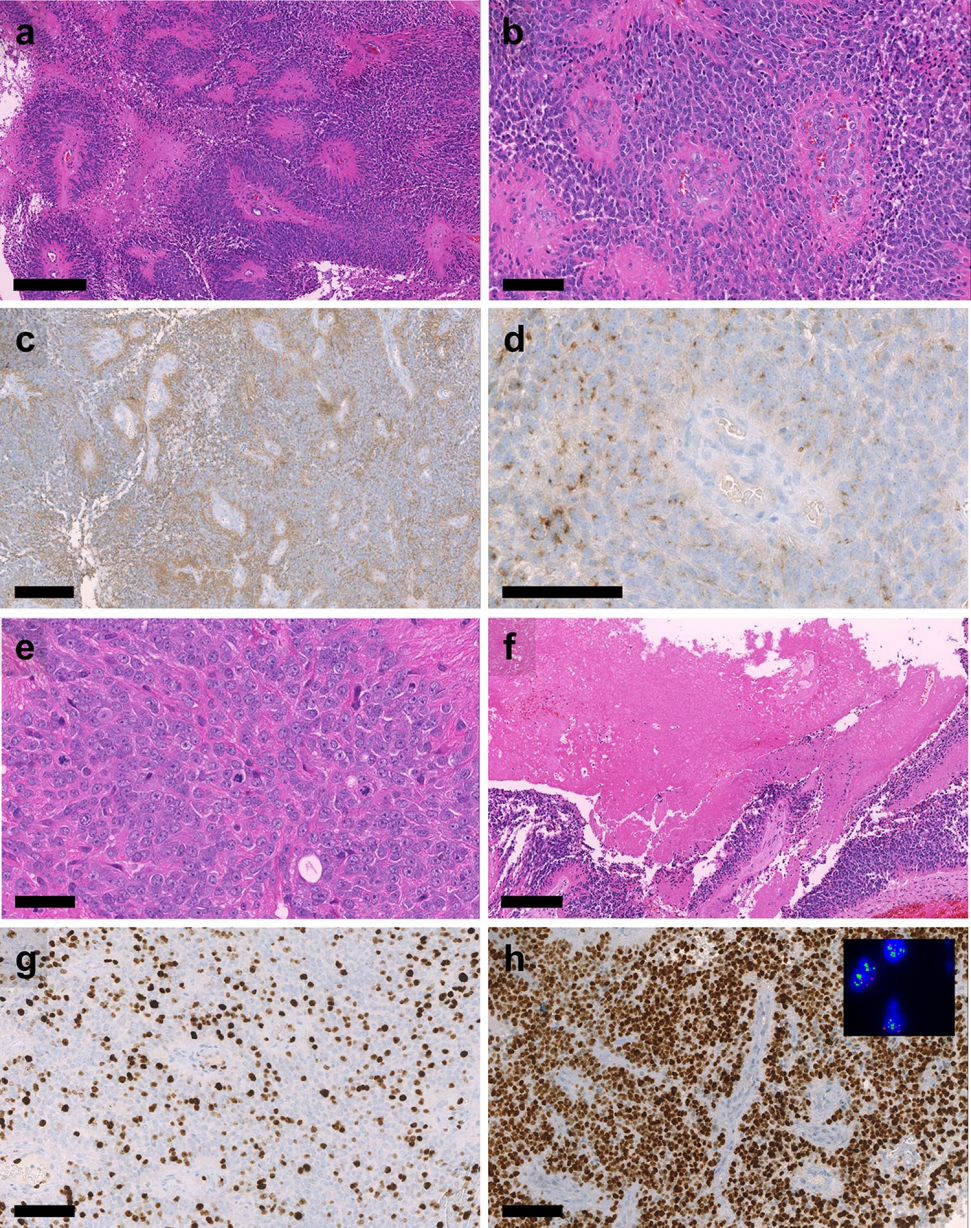
Histological features of SP-EPN-MYCN Highly cellular neuroectodermal tumor with **a** perivascular pseudorosettes, **b** microvascular proliferation, **c** perivascular enhanced GFAP expression, **d** dot-like EMA positivity, **e** brisk mitotic activity, **f** tumor necrosis, **g** high Ki-67 labelling, and **h** extensive nuclear MYCN expression. Inset in h): Results of two- color fluorescence in situ hybridization demonstrating multiple green signals for the *MYCN*-locus-probe and 2 red signals for the centromeric control probe. Scale bar = 300 µm in **a**, 100 µm in **b**, **d**, **g**, **h**, 200 µm in **c**, **f** and 50 µm in **e**

DNA methylation array-based CNV plots revealed focal high-level *MYCN* amplification for all 13 samples of the cohort (Fig. 1b, c, Suppl. Figure 1a, online resource) and several additional chromosomal aberrations at various frequencies, e.g. loss of chromosome 10 (3/13) or focal losses on Chromosome 11q (5/13) (Fig. 2d). Patient 1 additionally showed a *BRD4* amplification on chromosome 19p which was maintained throughout several relapses (Suppl. Figure 1b, online resource) and patient 6 showed an additional *YAP1* amplification on chromosome 11. Since *MYCN* amplification is characteristic for aggressive neuroblastomas which are often located close to the spine as well as for a distinct subset of pediatric glioblastomas [13, 21], we repeated DNA methylation-based clustering for the distinct spinal ependymoma cohort with two reference sets of 105 neuroblastomas and 11 MYCN-amplified pediatric glioblastomas, confirming the distinct methylation class of these cases (Suppl. Figure 2, online resource). Additionally, CNV plots were generated for six relapses (from patients 1 and 2). The *MYCN* amplification remained stable in all six relapsed cases (example given in Suppl. Figure 1b, online resource).

**Fig. 3:**
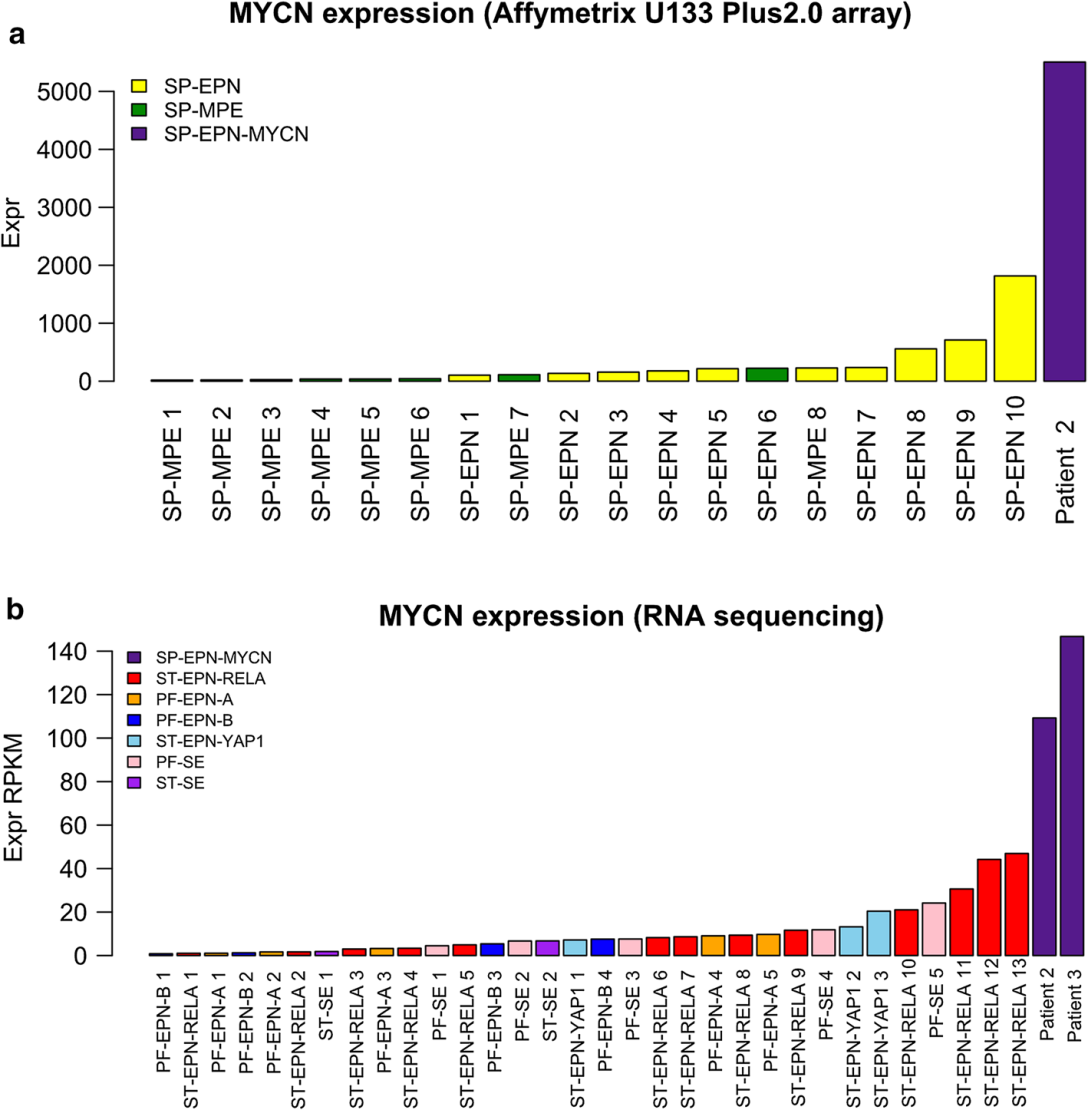
*MYCN* expression in SP-EPN-MYCN compared to other molecular ependymoma subgroup. **a** Relative level of *MYCN* expression in a sample from patient 2 was compared to *MYCN* expression in SP-MPE (*n* = 8) and SP-EPN (*n* = 10) (Affymetrix U133 Plus2.0 array data). **b***MYCN* expression in samples from patient 2 and 3 determined by RNA-sequencing compared to 34 samples representing all intracranial molecular subgroups of ependymoma (*n* = 34)

*MYCN* amplification was validated using fluorescence in situ hybridization (FISH) in cases for which FFPE tissue could be obtained (*n* = 5 primary tumors, *n* = 7 relapsed tumors). High-level *MYCN* amplification was confirmed in all available samples (Fig. 2h; Table 1). A limited set of RNA sequencing data (*n* = 2) and gene expression profiles generated on the Affymetrix U133 Plus2.0 array (*n* = 1) allowed comparison of *MYCN* expression levels with a cohort of spinal ependymal tumors (*n* = 18) comprising molecular subgroups SP-MPE (*n* = 8) and SP-EPN (*n* = 10) as well as to a cohort representing all intracranial molecular subgroups of ependymoma (*n* = 32) (Fig. 3a, b). *MYCN* amplified samples showed the highest expression of *MYCN* compared to both other cohorts. RNA sequencing did not provide significant evidence for additional genetic drivers, such as gene fusions (data not shown).

**Table 1:** Histological evaluation of all samples for which material was available

Patient	Specimen	Grade	Cellularity	Vessels	Mitotic activity	Necrosis	MYCN-FISH	MYCN-IHC	GFAP	EMA	H3K27me3	Ki67 up to (%)
1	Primary	II	Low	No MP	Low	No	Amp	++	+++	Dot-like	Retained	3
1	Relapse 1	III	Low and high	No MP	Brisk	No	Amp	++ to +++	+ with PE	Dot-like	Retained	20
1	Relapse 2	III	High	MP	Brisk	Present	Amp	+++	+ with PE	Dot-like	Retained	30
1	Relapse 3	III	High	MP	Brisk	Present	Amp	+++	+ with PE	Dot-like	Retained	20
1	Relapse 4	III	High	MP	Brisk	Present	Amp	+++	+ with PE	Dot-like	Retained	30
2	Primary	III	High	No MP	Moderate	No	Amp	+++	+++	Dot-like	Retained	15
2	Relapse 1	III	High	MP	Moderate	No	Amp	+++	+++	Dot-like	Retained	15
2	Relapse 2	III	Low and high	MP	Brisk	Present	Amp	++ to +++	++ with PE	Dot-like	Retained	25
2	Relapse 3	III	High	MP	Brisk	Present	Amp	+++	+ with PE	Dot-like	Retained	25
5	Primary	III	Low and high	MP	Brisk	No	Amp	++ to +++	++ with PE	Dot-like	Retained	30
10	Primary	III	Low and high	MP	Brisk	Present	Amp	++ to +++	++	Dot-like	Retained	60
12	Primary	III	Low and high	MP	Brisk	No	ND	++ to +++	++ with PE	Dot-like	Retained	40

*MP* microvascular proliferation, *PE* perivascular enhancement

+ focal, ++ weak to moderate and widespread, +++ strong and widespread

In conclusion, DNA methylation profiling identified a novel molecular group of spinal tumors with focal *MYCN* amplification that separates them from previously defined molecular subgroups of spinal ependymal tumors and that is also distinct from other tumor entities with *MYCN* amplification that may localize in or close to the spinal cord.

### Histopathological evaluation demonstrates ependymal differentiation and provides evidence for malignant progression

All 13 cases of our cohort were independently diagnosed as ependymoma by different neuropathologists at 11 centers in Europe, Australia, and North America. Cases were described as ependymoma, WHO Grade II (*n* = 3, including one tanycytic ependymoma) or anaplastic ependymoma, WHO Grade III (*n* = 10). Histopathological evaluation was complemented by electron microscopy for one sample of the cohort (patient 11) identifying intermediate filaments, ciliary structures and zipper-like tight junctions, which are classic ultrastructural features of ependymoma (Suppl. Figure 3, online resource) [17, 28].

For tumors where material was available (*n* = 12, five primaries and seven relapses), samples were re-evaluated by an experienced neuropathologist from our center (D.E.R.) confirming the initial diagnoses (Table 1). All tumors exhibited histological signs of ependymal differentiation with perivascular pseudorosettes, perivascular GFAP expression, and dot-like positivity for EMA. Microvascular proliferation was also frequently observed. (Fig. 2a–d, Table 1). Most tumors showed brisk mitotic activity and high Ki67 labelling indices (Fig. 2e and g). Tumor necrosis was present in most cases and in all late manifestations (Fig. 2e–g). H3K27me3, which is consistently lost in PF-EPN-A, was retained in all cases (Table 1). All tumors showed widespread expression of MYCN (Fig. 2h). One primary tumor did not show histological high-grade features, but its recurrence showed brisk mitotic activity and overall histological features of anaplasia, i.e. evidence of malignant progression (Suppl. Figure 4, online resource). Interestingly, while an *MYCN* amplification was detectable in both tumors, the intensity of the immunohistochemical MYCN expression was strongly increased in histological high-grade areas of the recurrent tumor while low-grade areas present in the same FFPE block still showed an only moderate MYCN labelling intensity. Different components with high- and low-grade morphology in tumors of the other patients also provided evidence for a malignant progression during the course of disease. The pattern of differential intensity of the immunohistochemical MYCN expression in histological low- and high-grade components in the same FFPE block was a consistent feature. A strong association between MYCN staining intensity and Ki67 labelling was evident (Table 1; Fig. 2). Taken together, this suggests that *MYCN* amplification is an early event in tumorigenesis and that malignant progression is associated with a further increase in MYCN protein levels.

**Fig. 4:**
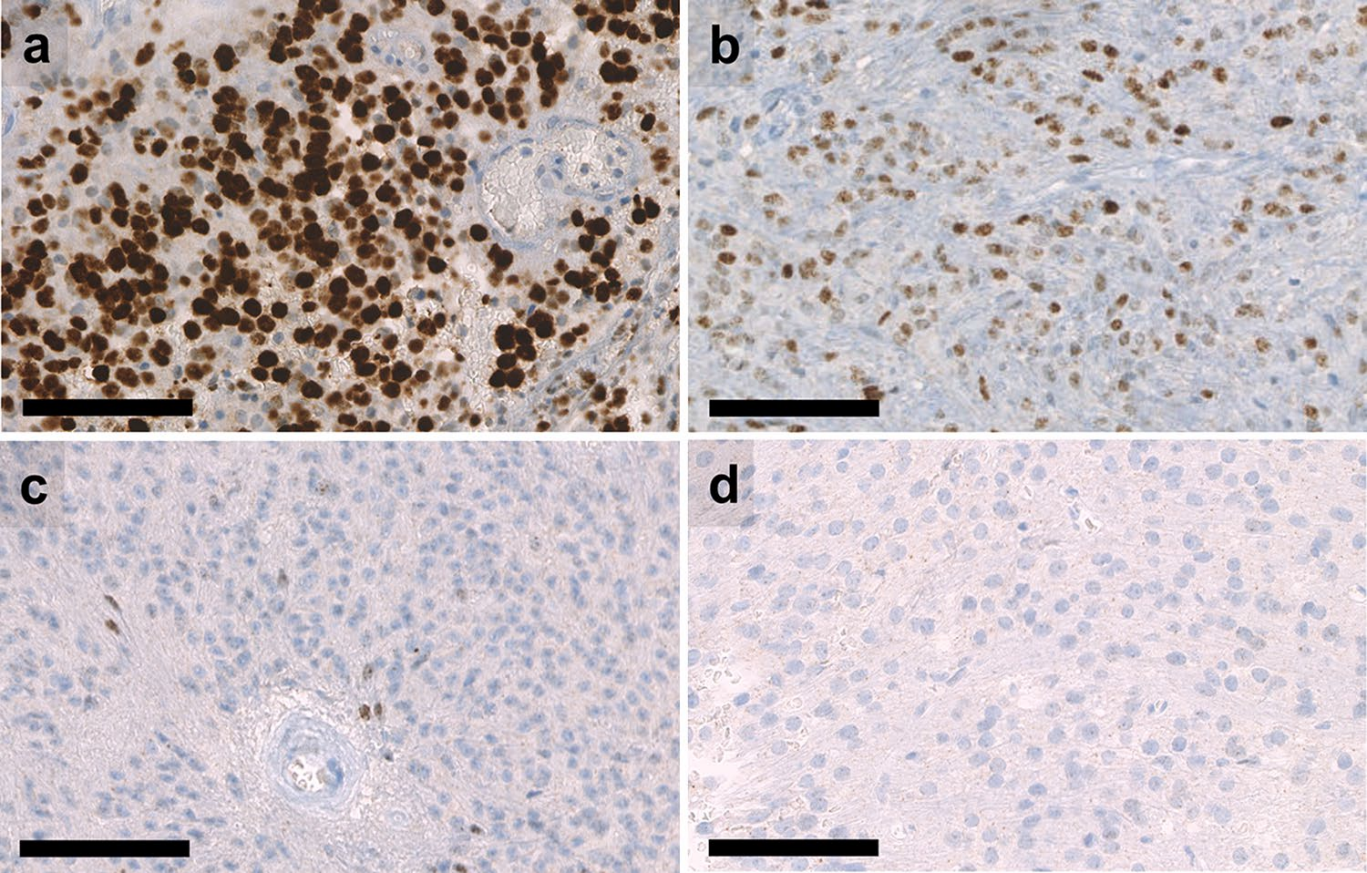
Immunohistochemical *MYCN* expression in spinal ependymomas. **a** Strong and widespread expression of MYCN in a SP-EPN-MYCN tumor that was diagnosed as grade III. **b** Moderate but widespread expression of MYCN in a SP-EPN-MYCN tumor that was diagnosed as grade II. **c** SP-EPN with sparse expression of MYCN in a few tumor cells. **d** Example of SP-EPN without expression of MYCN. Scale bar = 100 µm

Given that all tumors of the cohort showed widespread immunohistochemical MYCN expression, we evaluated whether immunohistochemical MYCN expression may serve as a surrogate marker for *MYCN* amplification. We stained 20 spinal ependymomas without *MYCN* amplification, 10 of the methylation group SP-EPN and 10 of the methylation group SP-MPE. None of these tumors showed a strong expression of MYCN. While the majority of cases showed no immunolabelling at all or only occasional positive cells, MYCN-amplified tumors showed a clearly distinct staining pattern with widespread and usually strong immunohistochemical MYCN expression (Fig. 4).

Considering these findings as well as the results of the molecular analysis, which showed *MYCN* amplification as the characteristic copy number alteration in these tumors, we suggest to designate this new subgroup “Spinal Ependymoma with *MYCN* amplification” (SP-EPN-MYCN).

### Demographic and radiological features of SP-EPN-MYCN

**Fig. 5:**
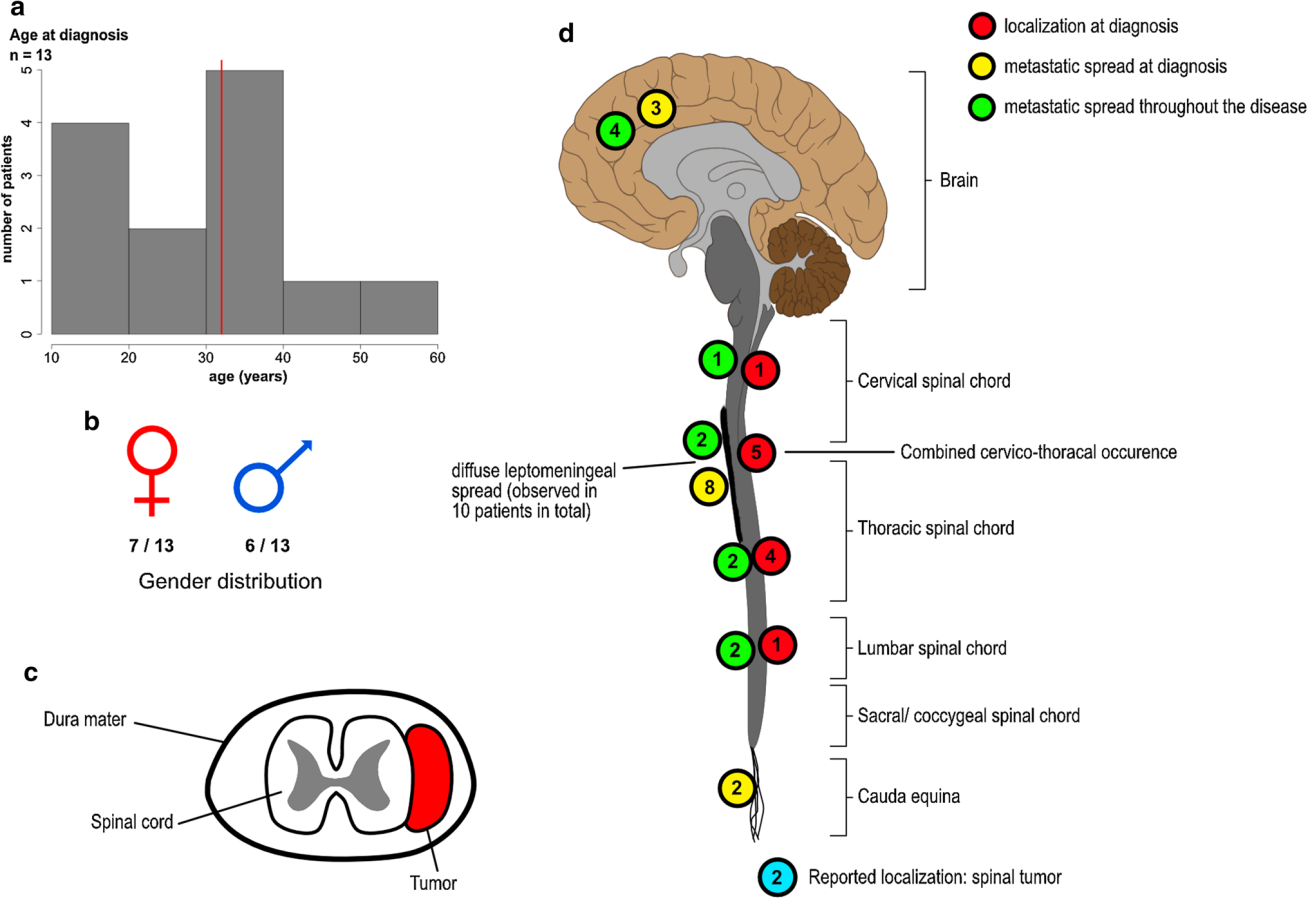
Clinicopathological variables of SP-EPN-MYCN. **a** Histogram depicting age of onset at first diagnosis. Red line marks the median (32 years). **b** Gender distribution was even with six male and seven female patients. **c** Schematic transversal depiction of the spinal cord showing extramedullar, intradural localization of tumors as reported in seven patients. **d** Localization of primary tumors and metastases throughout the CNS. Information regarding the localization of primary tumors and metastatic spread was available in 11/13 and 10/13 patients, respectively. Several patients showed multiple sites of metastatic spread. Localization of the primary lesion is shown in red, localization of metastases at diagnosis is shown in yellow, metastatic spread throughout the course of disease is shown in green. For two patients information regarding the localization at diagnosis was only given as “spinal” (blue circles)

**Fig. 6:**
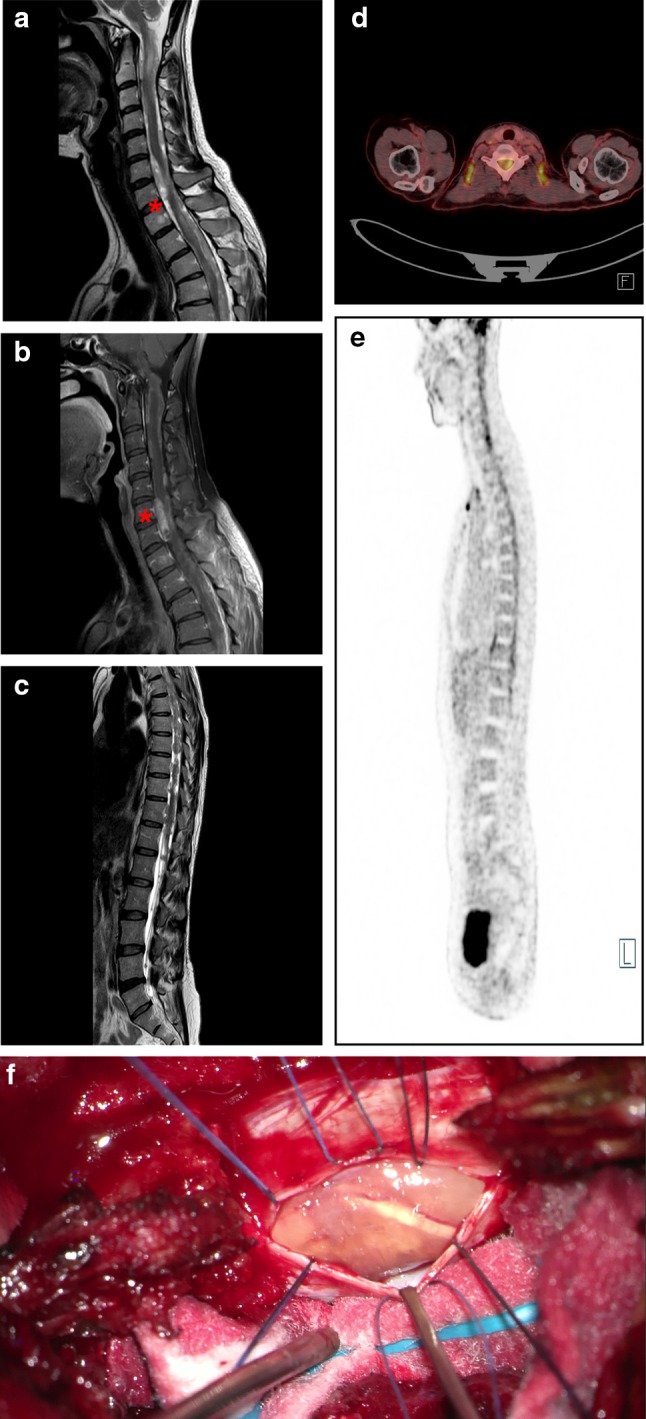
Radiological scans and intraoperative photograph of patient 12 **a** sagittal cervical MRI-T2 scan, red asterisk marks intradural, extramedullar tumor (C7) **b** sagittal cervical contrast enhanced MRI-T1 scan **c** sagittal MRI-T2 scan, the spinal canal is filled with widely disseminated tumor masses **d** and **e** F-18-FDG PET-CT (transversal, C7 (**d**) and sagittal, whole spine (**e**)) shows raised metabolism corresponding with the tumor at level C7 **f** intraoperative photograph taken during initial biopsy showing intradural mass

Demographic and basic radiological data were available for 13/13 and 11/13 of SP-EPN-MYCN tumors, respectively. Information regarding metastatic spread at diagnosis and during the course of disease could be obtained for 10/13 patients, respectively (Figs. 5 and 6, Suppl. Figure 5, online resource). Median age of onset was 32 years (range 12–56 years) and, therefore, lower than in previous studies on spinal ependymal tumors which reported a median age of about 40 years [7, 18] (Fig. 5a). Patient gender was evenly distributed across the cohort with seven female and six male cases (Fig. 5b). Next we asked whether there is a predilection site for SP-EPN-MYCN similar to SP-MPE and SP-EPN, which are mostly located at the conus medullaris/filum terminale and in the cervical/thoracic spine, respectively [7, 31]. Exact location in relation to the spinal meninges could be determined from radiological data of seven SP-EPN-MYCN patients. For all of these, the primary tumor was located intradurally and extramedullary (Fig. 5c). The majority of the cases arose in the cervical or thoracic spinal cord (*n* = 10), with only one case showing lumbar localization at initial presentation. Primary lesions were large, with only one case being limited to a single spinal segment. Nine out of ten cases showed multi-locular, diffuse leptomeningeal dissemination at diagnosis, including intracranial metastases in three cases. Diffuse leptomeningeal spread at some point throughout the course of disease was reported in all patients (10/10), including the two cases that did not show metastatic spread at first presentation. However, dissemination was not limited to the leptomeninges, but included nodular lesions as well. Cystic compartments within the malignant lesions were reported in four of the seven patients for whom radiological footage was available. Representative radiological images of an SP-EPN-MYCN tumor in a 46-year-old female patient are given in Fig. 6 (see also: Suppl. Figure 5, online resource). In conclusion, SP-EPN-MYCN tumors were mainly diagnosed in adolescence and early adulthood and showed distinct radiological features, including extramedullary location and diffuse leptomeningeal spread, thus differing strongly from previously described spinal ependymoma cases [37, 45].

### SP-EPN-MYCN show dismal outcome despite high intensity treatment

**Fig. 7:**
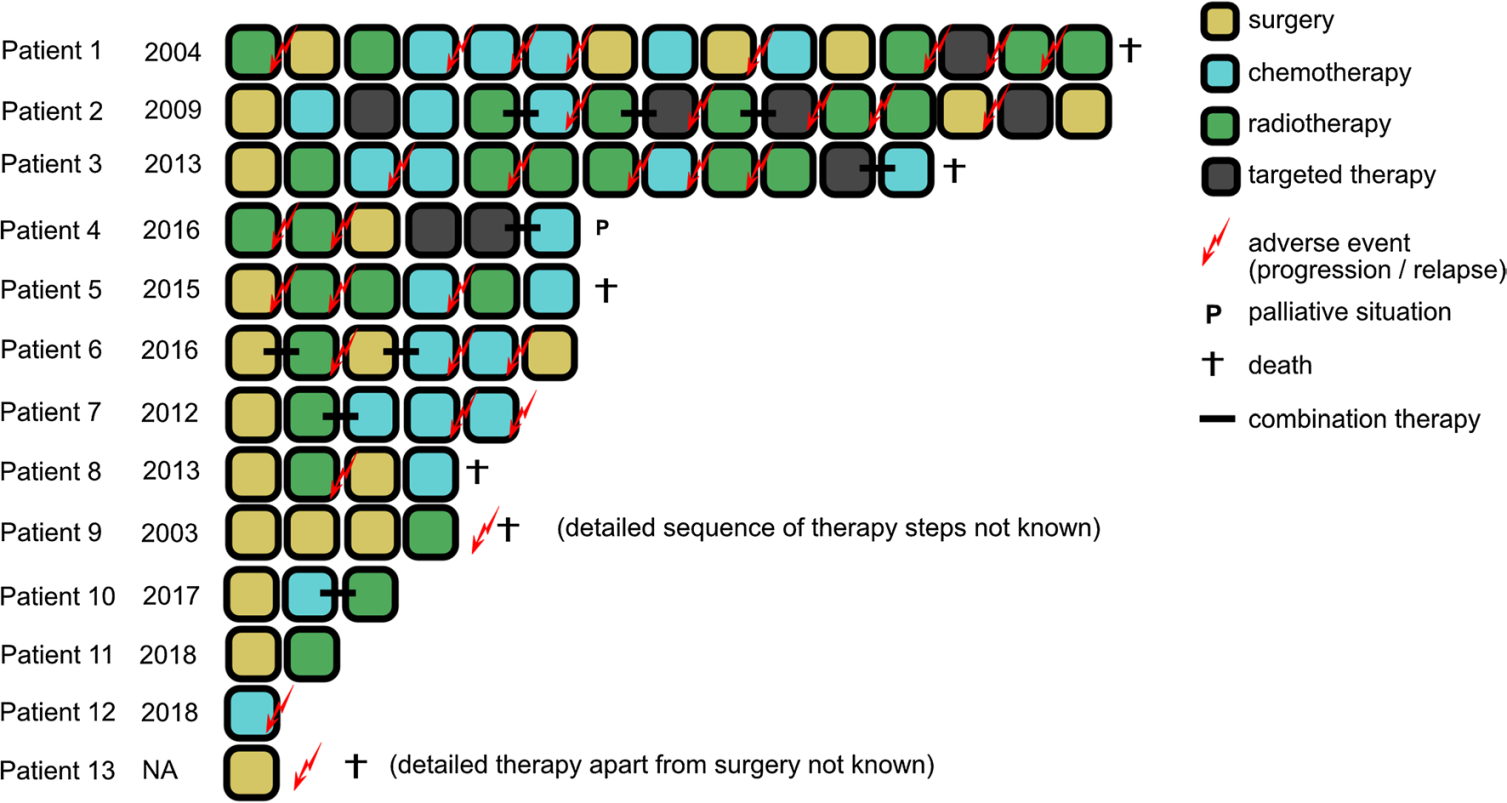
Visualization of therapy regimens used in individual patients of the SP-EPN-MYCN cohort Each box represents one therapeutic intervention, boxes connected with a black line were given as combination treatment. The sequence of the boxes represents the chronological order of therapeutic steps, apart from patient 9 for whom only limited data was available and therefore the exact chronological order of therapy events could not be reconstructed. The date indicates the year of the initial diagnosis. Biopsies were not counted as therapeutic interventions and are not listed, but took place in every patient (See also: Supp. Table 1)

**Fig. 8:**
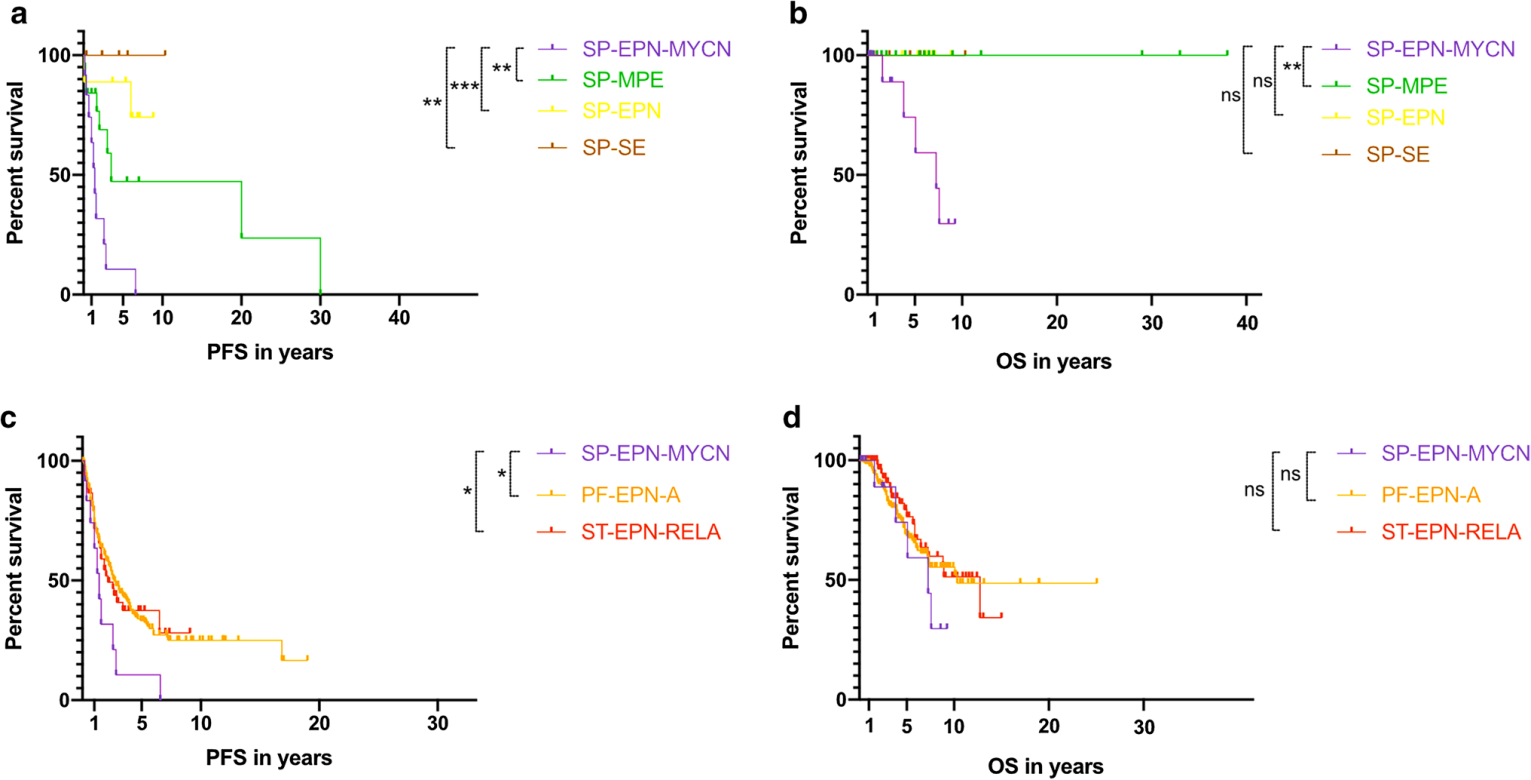
PFS and OS of SP-EPN-MYCN (*n* = 12) analyzed using Kaplan–Meier curves. **a** PFS and **b** OS of SP-EPN-MYCN were compared with all other molecular spinal ependymal subgroups as well as **c**, **d** ST-EPN-RELA and PF-EPN-A. P values were calculated using log-rank-tests between the molecular subgroups. ns = *p*  > 0.05, * = *p* < 0.05, ** = *p* ≤ 0.01, *** = *p* ≤ 0.001. No survival data was available for patient 13. (Pajtler et al. 2015; Kraetzig et al. 2018)

Detailed clinical data were collected and subsequently analyzed in 12 of 13 cases. The SP-EPN-MYCN cohort showed aggressive behavior, including early metastases, rapid progression after relapse, dissemination throughout the whole CNS, and resistance to common treatment strategies. Successful gross total resection of the primary tumor was reported for one patient only (1/12) but could not be achieved in others (11/12) due to metastatic spread at diagnosis (9/12) or extended lesions that would have resulted in non-acceptable side effects from surgery (2/12). In the majority of cases, surgery was the initial therapeutic step (9/12) (Fig. 7). All patients relapsed or progressed, often with metastatic spread, at some point during the course of disease. Figure 7 and Suppl. Table 1 summarize applied treatment strategies for all cases. Despite highly intensive treatment regimens, including repeated surgery, radiotherapy, different chemotherapy protocols, and targeted therapy, six patients were deceased and one was at a terminal disease stage at time of data collection. Of the remaining six cases, two were diagnosed in late 2018; thus, data on follow-up are limited. Chemotherapy was applied as single agent or combinatorial treatment including temozolomide, carboplatin, etoposide/cyclophosphamide, etoposide/carboplatin, vincristine/cyclophosphamide, and trofosfamide. In one patient, Imatinib was used due to high c-Kit expression of the tumor cells. Figure 8 shows survival of SP-EPN-MYCN (*n* = 12) compared to a reference set of cases of the three other molecularly defined spinal ependymal subgroups (Fig. 8a, b) as well as ST-EPN-RELA and PF-EPN-A (Fig. 8c, d). As reference sets for the subgroups SP-SE (*n* = 5), SP-EPN (*n* = 9), ST-EPN-RELA (*n* = 76), and PF-EPN-A (*n *= 219), data published by Pajtler et al. in 2015 [31] were used. For myxopapillary ependymoma (*n* = 19), a histologically defined reference set from Kraetzig et al. 2018 [22], was used due to a lack of sufficient numbers for molecularly defined SP-MPE with clinical data. Notably, a limited set of clinical data on molecularly defined SP-MPE that was not included showed identical clinical outcomes to histologically classified myxopapillary ependymoma from Kraetzig et al. 2018 [31]. The median PFS for SP-EPN-MYCN was 17 months and was significantly worse than for SP-SE (*p* = 0.006), SP-EPN (*p* = 0.001) and SP-MPE (*p* = 0.008) (Fig. 8a). No disease-related death was reported for any of the other spinal subgroups, whereas SP-EPN-MYCN showed a median OS of 87 months and dismal outcome compared with SP-SE (*p* = 0.166), SP-EPN (*p* = 0.050), and SP-MPE (*p* = 0.005) (Fig. 8b).

Since spinal ependymal tumors are known to have a relatively benign prognosis in general, we also compared SP-EPN-MYCN tumors with ST-EPN-RELA, and PF-EPN-A, as these two subgroups show the worst prognosis of all so far described molecular ependymoma subgroups [31]. There was no significant difference in OS between SP-EPN-MYCN and ST-EPN-RELA (*p* = 0.252) or PF-EPN-A (*p* = 0.353) (Fig. 8c). Notably, PFS was significantly worse in SP-EPN-MYCN compared with PF-EPN-A (*p* = 0.017), and ST-EPN-RELA (*p* = 0.047) (Fig. 8d).

In conclusion, SP-EPN-MYCN showed significantly reduced PFS and OS compared to all other spinal ependymoma entities and similar prognosis as unfavorable intracranial subgroups ST-EPN-RELA and PF-EPN-A, thus confirming the highly aggressive nature of this newly defined molecular subgroup of spinal ependymal tumors.

## Discussion

In this study, we identified and characterized a novel molecular subgroup of spinal ependymoma designated as SP-EPN-MYCN with histological features of ependymoma and a characteristic *MYCN* amplification. *MYCN* belongs to the family of *MYC*-oncogenes and is an important regulatory gene during neuronal embryogenesis [19]. It plays a major role in the formation and proliferation of a wide range of malignancies, including neuroblastoma, pediatric glioblastoma, medulloblastoma, nephroblastoma, leukemia, prostate cancer, and lung cancer, and often correlates with poor prognosis [3, 4, 10, 21, 23, 32, 34, 42]. Scheil et al. reported on two cases of spinal *MYCN*-amplified ependymoma staged as WHO Grade II and WHO Grade III, respectively [35]. Both tumors showed histopathological patterns that were similar to cases described in our study. For one of these cases, relapse, metastatic spread into the subarachnoid space and intracranially was documented. Notably, the WHO Grade II tumor in their study showed the same pattern of malignant progression at relapse as those in our study [35]. Interestingly, their second case was also characterized by multiple schwannomas, indicating a possible NF2-syndrome in the affected patient. Patients described in our study did not show any signs related to NF2 and no NF2 mutations were found in both patients for whom RNA-sequencing was available. One patient was diagnosed with an embryonal testicular tumor 2 years before the detection of the spinal ependymoma. There is no clear evidence for a correlation of SP-EPN-MYCN with a cancer predisposition syndrome, although the cohort is too small to finally answer this question. Since we could not detect any other major genetic aberration apart from a *YAP1* amplification and a *BRD4* amplification each in one case, we strongly assume that *MYCN* amplification is the main driving oncogenic event in SP-EPN-MYCNs as is also known for aggressive neuroblastoma, in which *MYCN* amplification is sufficient for tumor formation and progression [41]. This hypothesis is further supported by the fact that MYCN amplification was retained in all relapses and by a recurrent immunohistochemical MYCN expression pattern suggesting malignant progression being associated with a further increase in MYCN abundance. Notably, it was previously shown that transcription of *MYCN* in *MYCN* amplified neuroblastoma cells relies on *BRD4* expression and that *BRD4* inhibition by BET-bromodomain inhibitors decreases MYCN expression [14, 33].

In contrast to SP-EPN that are mostly located in the center of the spinal cord [37, 38], all cases for which we were able to obtain detailed information were located intradural and extramedullary. The distinct methylation patterns of the different spinal ependymoma subgroups may be indicative of dissimilar cells of origin. Intradural, extramedullary ependymoma apart from SP-MPE is very rare and only sparse information regarding these tumors is available in the literature. Several case reports describe patients that showed similar features as SP-EPN-MYCN, such as multilocular disease, diffuse leptomeningeal spreading and characteristic ependymal histological features [11, 16, 36]. Notably, a previous study reports on a pediatric case in which nodular lesions were detectable in an MRI scan 2 years prior to onset of disease-related symptoms [36]. Their review of the literature revealed 24 cases of intradural, extramedullary ependymomas with varying outcome but common epidemiological and radiological features, i.e. localization within the cervical/thoracic spine, occurrence in adulthood and a higher prevalence in females. While several of the reported cases might represent SP-EPN-MYCN tumors with poor prognosis, others showed different characteristics, suggesting that rare cases of intradural extramedullary ependymoma with favorable outcome may exist apart from the molecular subgroups SP-MPE and SP-EPN-MYCN.

While the majority of spinal ependymomas are relatively benign and show favorable outcome, several epidemiological studies in the past reported a subset of patients with highly aggressive disease and poor survival [7, 18]. These cases were often diagnosed as anaplastic ependymoma WHO Grade III and it is likely that these represented tumors of the SP-EPN-MYCN subgroup. However, in our study three cases were initially diagnosed as ependymoma WHO Grade II, and all these tumors showed malignant progression histologically and clinically. We, therefore, propose that all cases of spinal ependymoma should be analyzed for the presence of an *MYCN* amplification, especially if they show disseminated disease at diagnosis. Based on our data, DNA methylation analysis is a suitable diagnostic tool which also supports unambiguous molecular classification [6]. IHC stainings for MYCN might serve as a suitable pre-screening method. In case SP-EPN-MYCN is diagnosed, a cranial MRI should be performed to exclude intracranial metastases as these were seen in three patients of our cohort at initial diagnosis. Close radiological monitoring also seems appropriate due to the fact that newly emerging metastases tended to show rapid growth.

We suggest to establish SP-EPN-MYCN as an additional major molecular subgroup of ependymoma. Given its clear diagnostic criteria and clinical impact SP-EPN-MYCN should also be in included in the next update of the WHO classification of tumors of the CNS. Regarding the devastating outcome of SP-EPN-MYCN, there is an urgent need for innovative therapeutic concepts for these patients. Due to the small patient cohort and the wide range of therapeutic strategies, it is not possible to draw conclusions regarding optimal treatment for SP-EPN-MYCN from our study. Although MYCN is currently still seen as an undruggable target, several strategies of MYCN-inhibition are currently being investigated. Amongst them, HDAC inhibitors, PARP inhibitors, Aurora A-kinase inhibitors and BET-bromodomain inhibitors are seen as promising candidates for translation into the therapy for *MYCN*-amplified tumors [4, 8, 12, 14, 33, 40]. There is also evidence that MYCN might be targetable through immunotherapy [39]. Whether one of these strategies is suitable for treating SP-EPN-MYCN tumors remains to be seen and should be investigated in future clinical trials.

## Electronic supplementary material

Below is the link to the electronic supplementary material.

Supplementary material 1 (TIFF 3935 kb). *MYCN* amplification as a characteristic CNV in SP-EPN-MYCN **a** CNV-plots of all 13 cases showing focal high-level *MYCN* amplification on chromosome 2p as characteristic copy number event in SP-EPN-MYCN

Supplementary material 2 (TIFF 1313 kb). *MYCN* amplification as a characteristic CNV in SP-EPN-MYCN. **b** CNV-plots of the primary tumor and three relapses of patient 1. While the *MYCN* amplification is conserved in all relapses, several new complete or partial gains and losses are observed in the relapse samples. A *BRD4* amplification on chromosome 19p remains stable throughout all relapses

Supplementary material 3 (TIFF 245 kb). SP-EPN-MYCN methylation patterns are distinct from other *MYCN* amplified entities T-SNE plot depicting unsupervised DNA methylation based clustering of SP-EPN-MYCN samples with a reference cohort of **a** 105 neuroblastomas (both *MYCN* amplified and *MYCN* non-amplified cases) and **b** 11 *MYCN* amplified pediatric high grade gliomas confirms SP-EPN-MYCN as distinct molecular group different other highly *MYCN*-amplified nervous system tumors. (Used data sets for reference cohorts: Henrich et al., Cancer Research, 2016 and Korshunov et al., Acta Neuropathologica, 2017)

Supplementary material 4 (TIFF 2143 kb). Ultrastructural features of ependymoma in SP-EPN-MYCN Electron microscopy of patient 11 shows sheets of cells with intermediate filaments, cilia and intercellular, frequently long, and zipper-like tight junctions. The case was histologically diagnosed as tanycytic ependymoma. Red arrows: cilia; asterisk: tight junctions

Supplementary material 5 (TIFF 16989 kb). Histological progression in SP-EPN-MYCN **a, c, e, g**: initial tumor of patient 1 showing **a** HE staining of tanycytic ependymoma grade II with **c** strong GFAP expression, **e** low Ki-67 labelling and **g** weak to moderate *MYCN* expression. **b, d, f, h:** recurrent tumor showing **b** hypercellularity, **d** reduced GFAP expression, **f** high Ki-67 labelling, and **h** extensive MYCN positivity. *MYCN* amplified nuclei demonstrated by FISH are present in both tumor manifestations (insets in **g** and **h**). Scale bar = 100 µm

Supplementary material 6 (TIFF 1943 kb). Radiological scans of SP-EPN-MYCN patients Scans from patient 5 (a, b) and patient 11 (c) **a** initial diagnosis of patient 5: sagittal lumbar MRI-T2 and contrast-enhanced MRI-T1, axial lumbar MRI-T2 and contrast-enhanced MRI-T1 at the level of L5 showing an intradural mass with multiple nodular leptomeningeal metastases **b** first recurrence of disease in patient 5: sagittal lumbar MRI-T2 and contrast-enhanced MRI-T1, axial lumbar MRI-T2 and contrast-enhanced MRI-T1 at the level of L5 showing tumor recurrence at the level of L5 and progressing leptomeningeal metastasis at the level of L2. Red asterisk = L5 **c** initial diagnosis of patient 11: sagittal MRI-T2 and MRI-STIR showing extensive leptomeningeal disease throughout the cervical and thoracic spinal canal

Supplementary material 7 (DOCX 14 kb). Summary of the histological, radiological, and clinical features of each patient
