# Supplementary figures and images for: MYCN amplification drives an aggressive form of spinal ependymoma
Source: Acta Neuropathol. 2019 Aug 14;138(6):1075–89. doi: 10.1007/s00401-019-02056-2 (PMC6851394; doi:10.1007/s00401-019-02056-2)

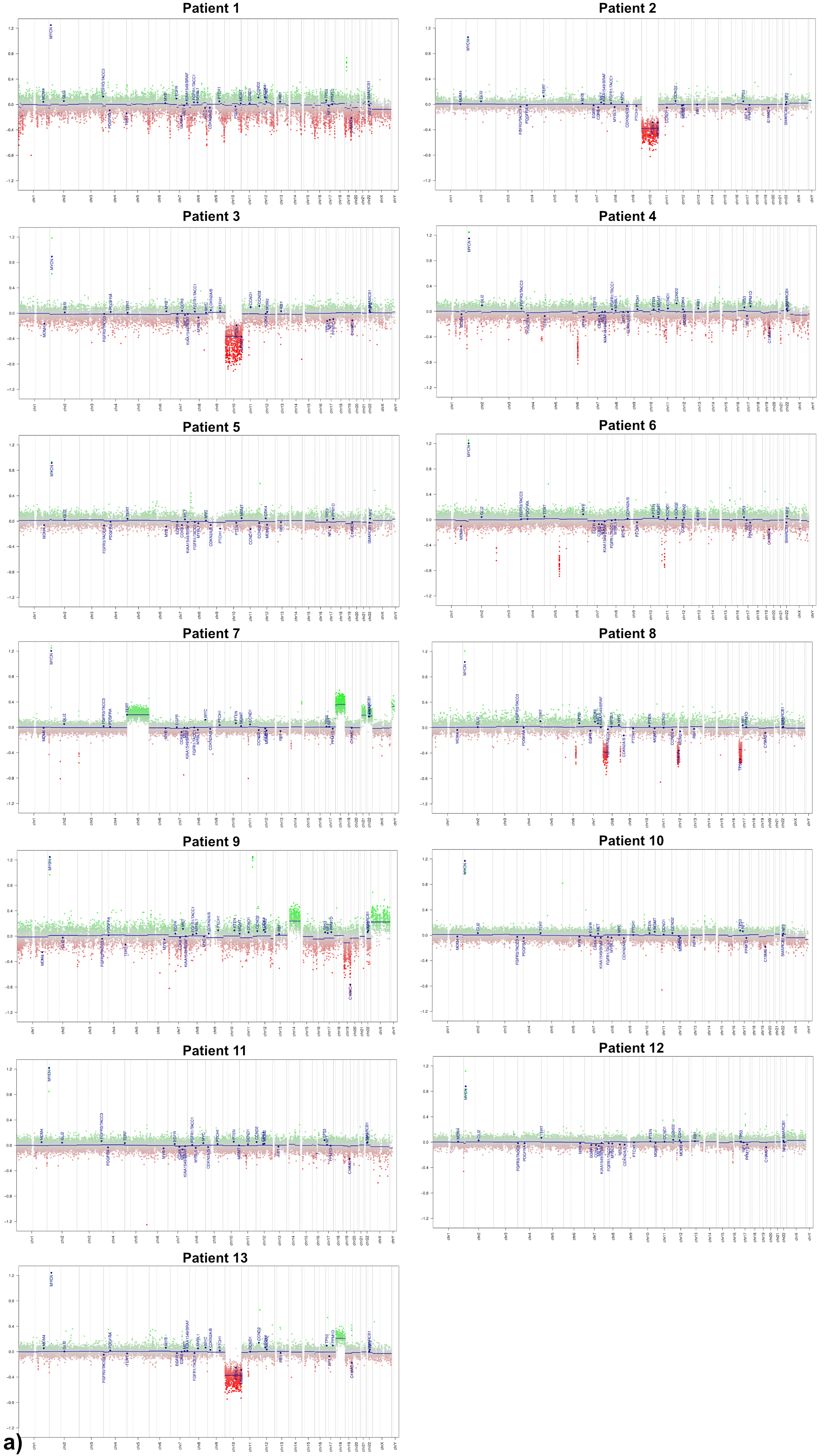

Supplement: Supplementary file 1 — Supplementary material 1 (TIFF 3935 kb). MYCN amplification as a characteristic CNV in SP-EPN-MYCN a CNV-plots of all 13 cases showing focal high-level MYCN amplification on chromosome 2p as characteristic copy number event in SP-EPN-MYCN [file 401_2019_2056_MOESM1_ESM.tiff]

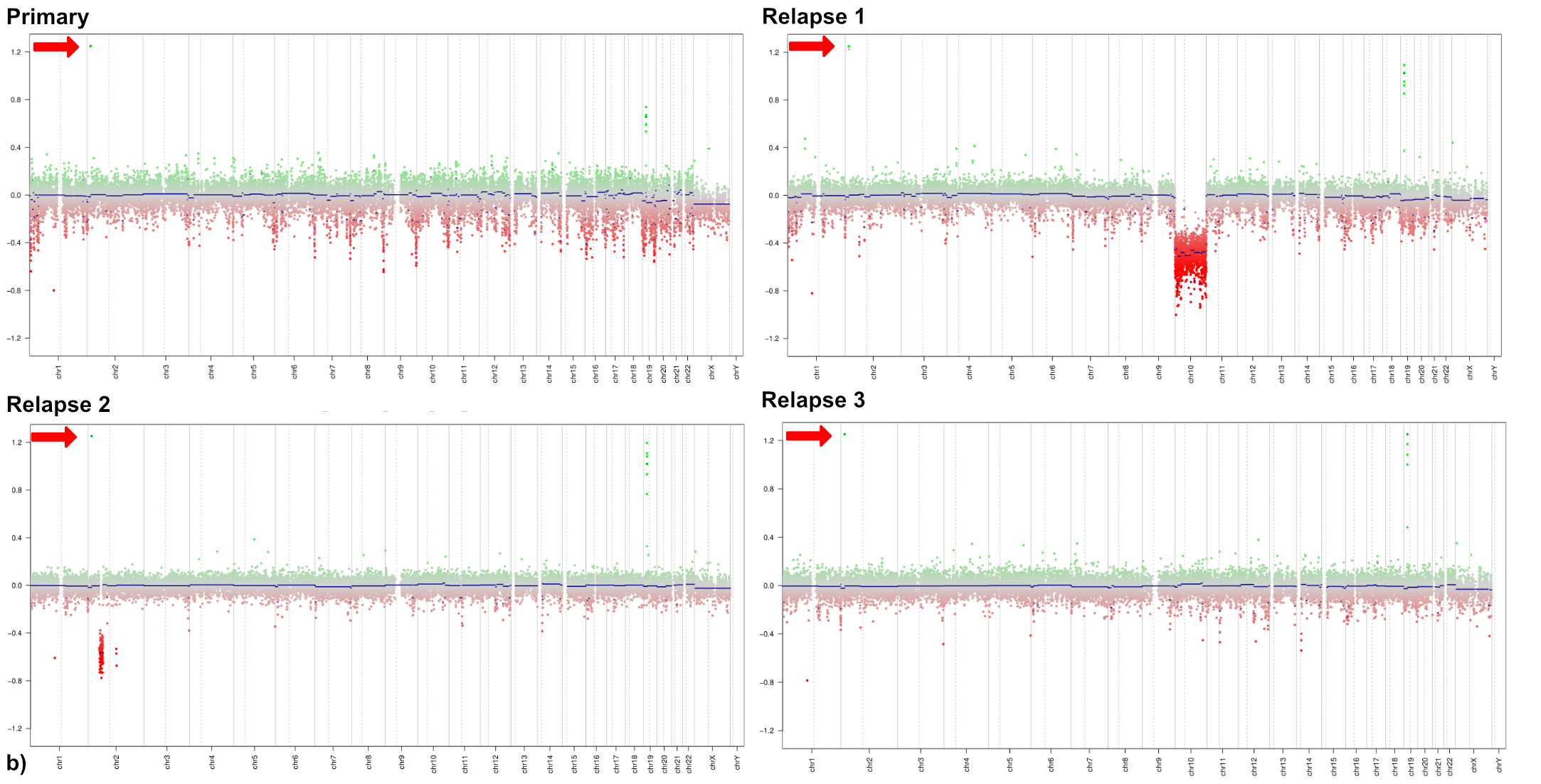

Supplement: Supplementary file 2 — Supplementary material 2 (TIFF 1313 kb). MYCN amplification as a characteristic CNV in SP-EPN-MYCN. b CNV-plots of the primary tumor and three relapses of patient 1. While the MYCN amplification is conserved in all relapses, several new complete or partial gains and losses are observed in the relapse samples. A BRD4 amplification on chromosome 19p remains stable throughout all relapses [file 401_2019_2056_MOESM2_ESM.tiff]

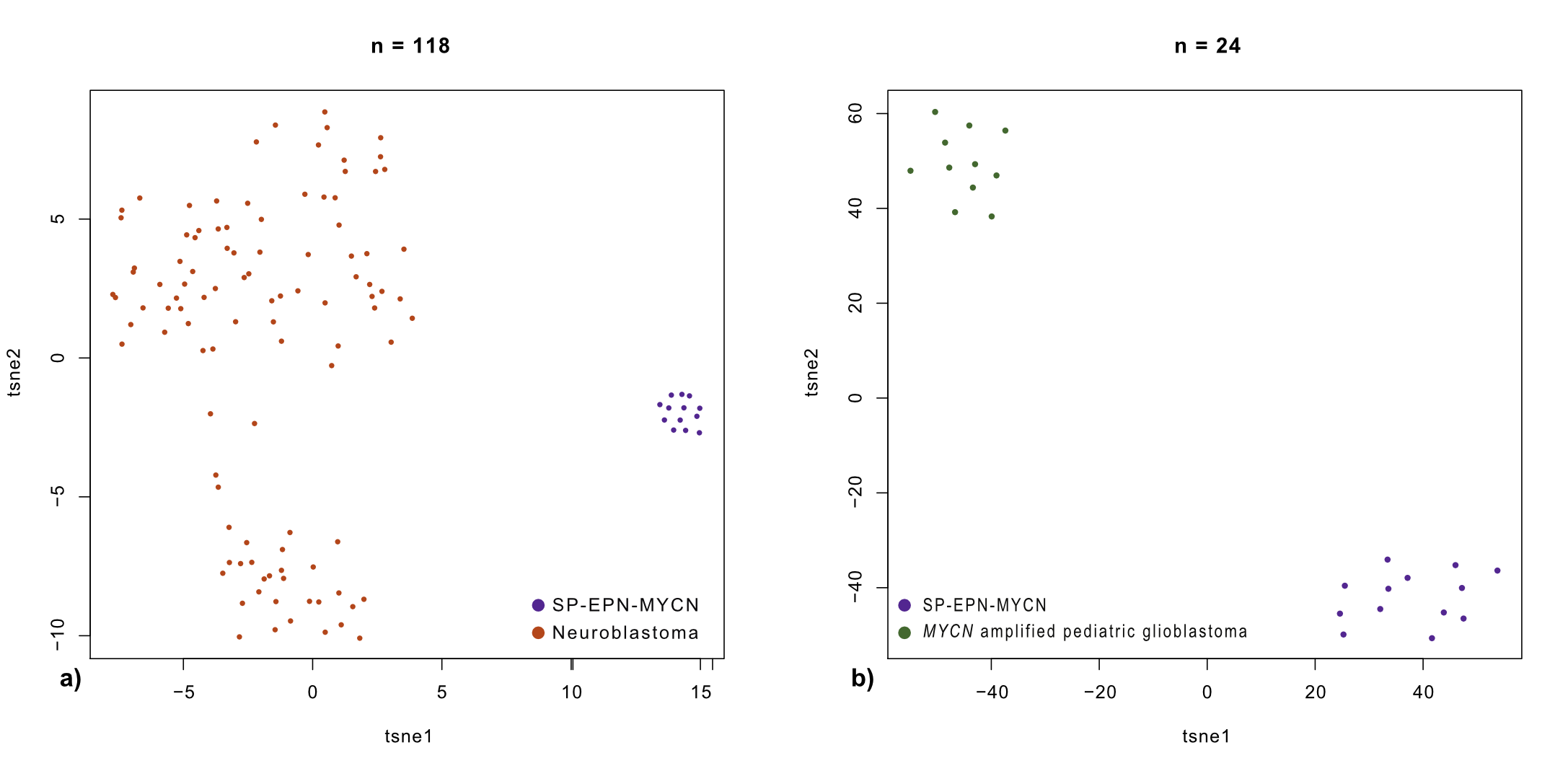

Supplement: Supplementary file 3 — Supplementary material 3 (TIFF 245 kb). SP-EPN-MYCN methylation patterns are distinct from other MYCN amplified entities T-SNE plot depicting unsupervised DNA methylation based clustering of SP-EPN-MYCN samples with a reference cohort of a 105 neuroblastomas (both MYCN amplified and MYCN non-amplified cases) and b 11 MYCN amplified pediatric high grade gliomas confirms SP-EPN-MYCN as distinct molecular group different other highly MYCN-amplified nervous system tumors. (Used data sets for reference cohorts: Henrich et al., Cancer Research, 2016 and Korshunov et al., Acta Neuropathologica, 2017) [file 401_2019_2056_MOESM3_ESM.tiff]

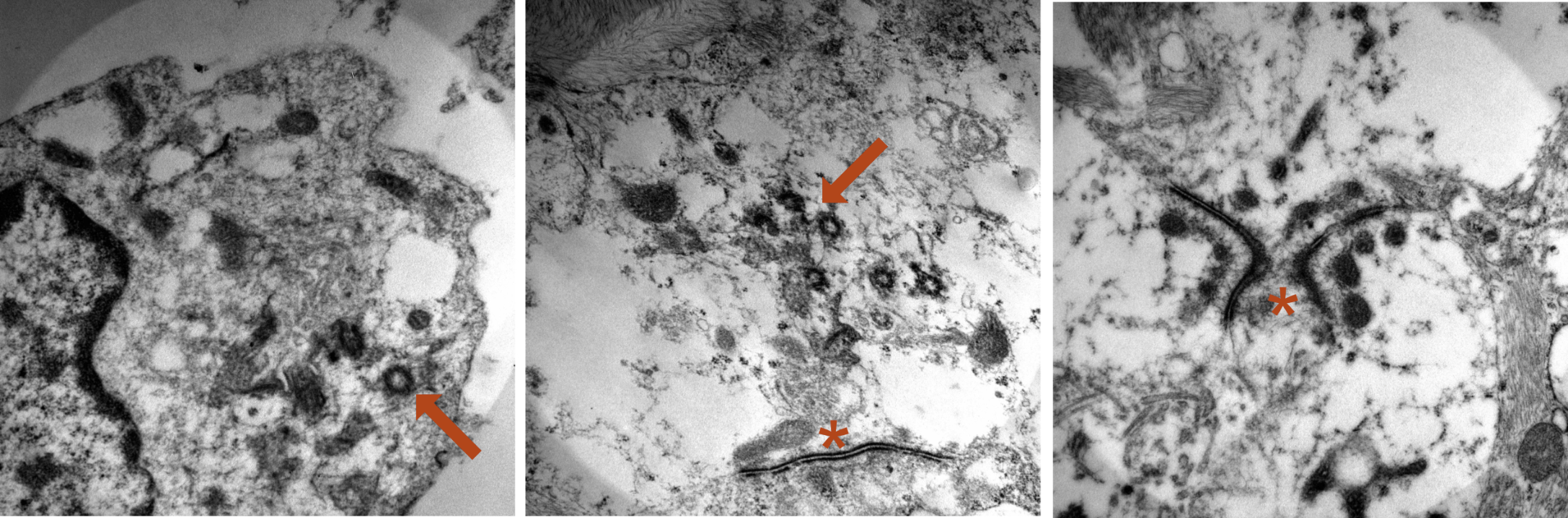

Supplement: Supplementary file 4 — Supplementary material 4 (TIFF 2143 kb). Ultrastructural features of ependymoma in SP-EPN-MYCN Electron microscopy of patient 11 shows sheets of cells with intermediate filaments, cilia and intercellular, frequently long, and zipper-like tight junctions. The case was histologically diagnosed as tanycytic ependymoma. Red arrows: cilia; asterisk: tight junctions [file 401_2019_2056_MOESM4_ESM.tiff]

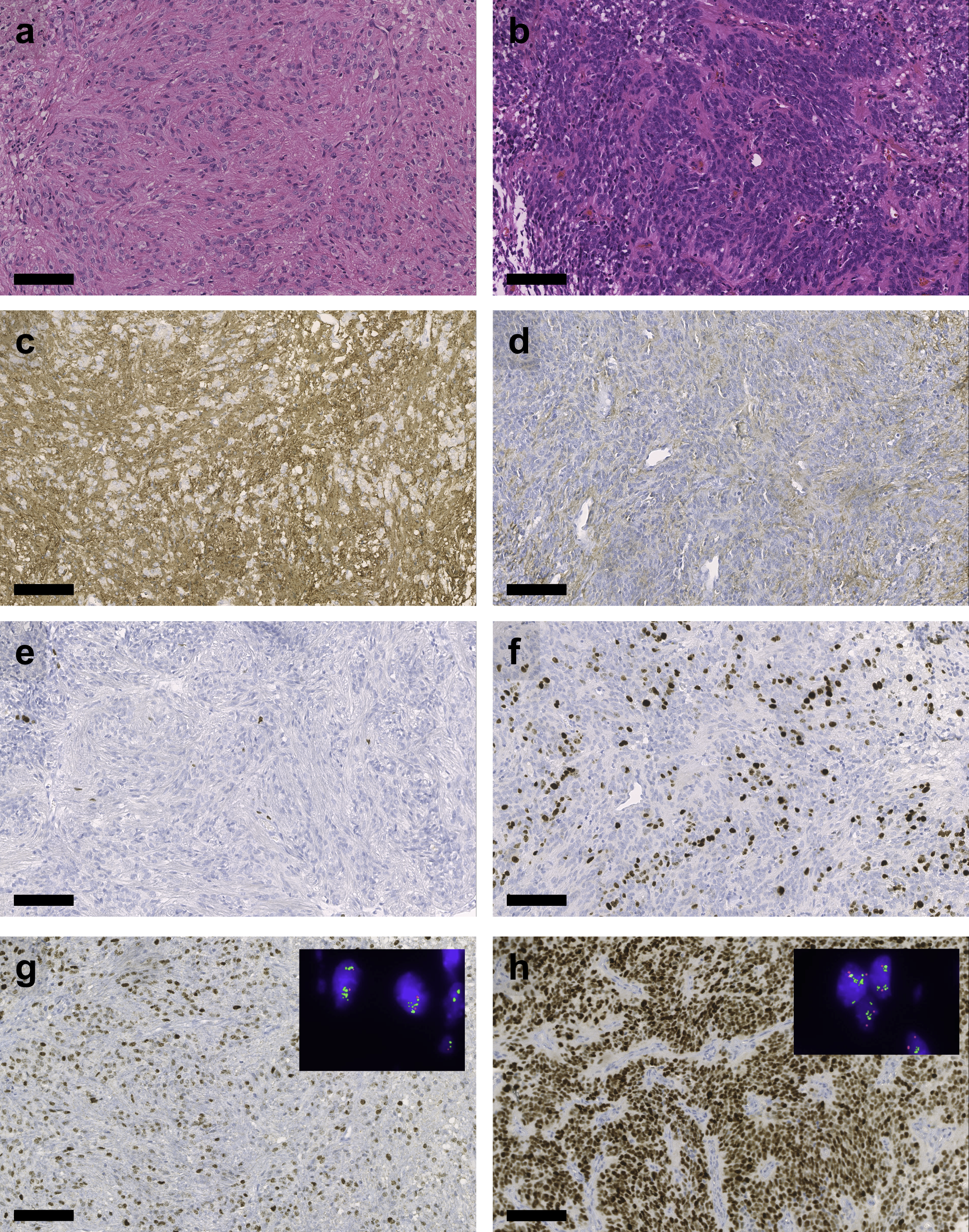

Supplement: Supplementary file 5 — Supplementary material 5 (TIFF 16989 kb). Histological progression in SP-EPN-MYCN a, c, e, g: initial tumor of patient 1 showing a HE staining of tanycytic ependymoma grade II with c strong GFAP expression, e low Ki-67 labelling and g weak to moderate MYCN expression. b, d, f, h: recurrent tumor showing b hypercellularity, d reduced GFAP expression, f high Ki-67 labelling, and h extensive MYCN positivity. MYCN amplified nuclei demonstrated by FISH are present in both tumor manifestations (insets in g and h). Scale bar = 100 µm [file 401_2019_2056_MOESM5_ESM.tiff]

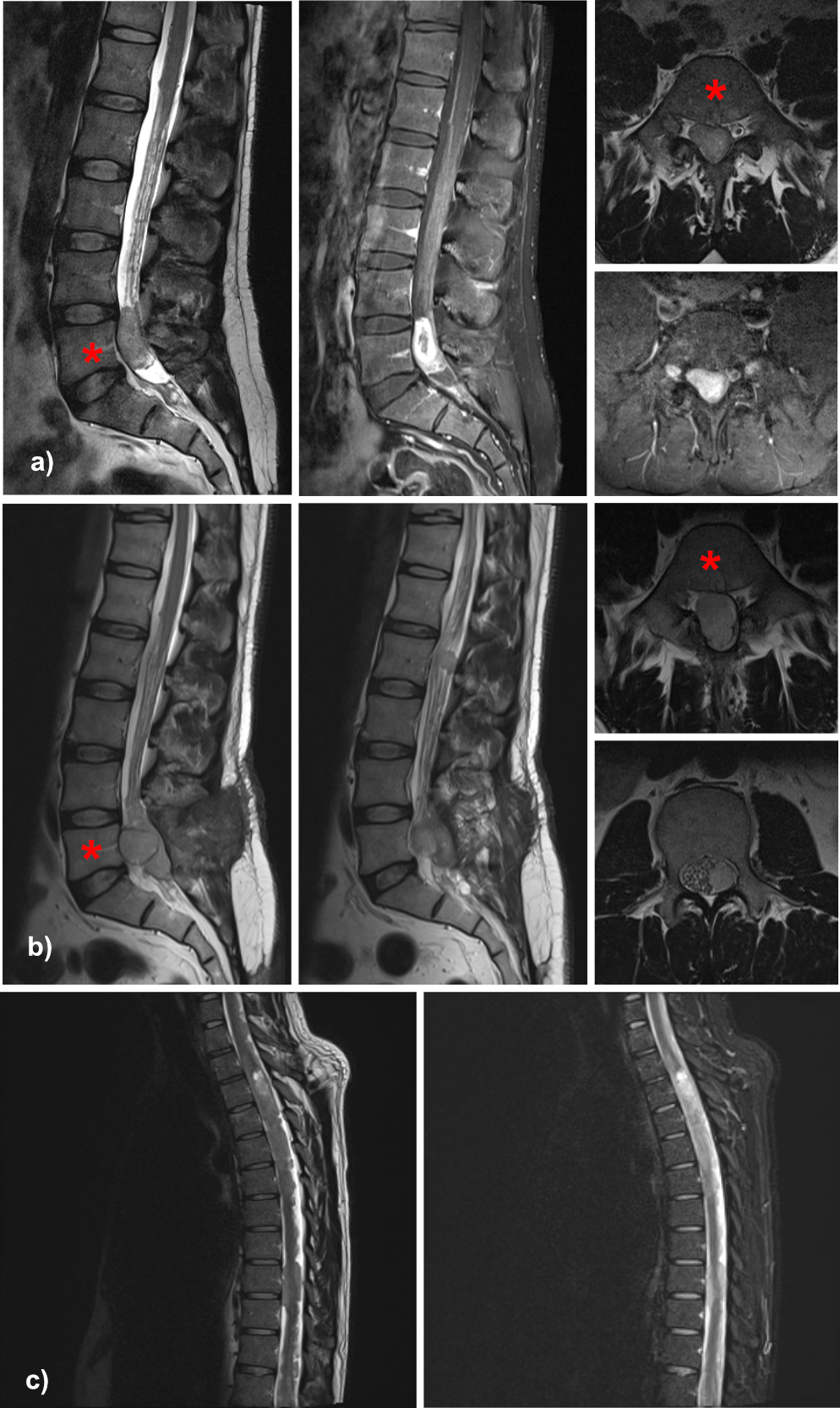

Supplement: Supplementary file 6 — Supplementary material 6 (TIFF 1943 kb). Radiological scans of SP-EPN-MYCN patients Scans from patient 5 (a, b) and patient 11 (c) a initial diagnosis of patient 5: sagittal lumbar MRI-T2 and contrast-enhanced MRI-T1, axial lumbar MRI-T2 and contrast-enhanced MRI-T1 at the level of L5 showing an intradural mass with multiple nodular leptomeningeal metastases b first recurrence of disease in patient 5: sagittal lumbar MRI-T2 and contrast-enhanced MRI-T1, axial lumbar MRI-T2 and contrast-enhanced MRI-T1 at the level of L5 showing tumor recurrence at the level of L5 and progressing leptomeningeal metastasis at the level of L2. Red asterisk = L5 c initial diagnosis of patient 11: sagittal MRI-T2 and MRI-STIR showing extensive leptomeningeal disease throughout the cervical and thoracic spinal canal [file 401_2019_2056_MOESM6_ESM.tiff]
