# Supplementary material for: MYCN amplification drives an aggressive form of spinal ependymoma
Source: Acta Neuropathol. 2019 Aug 14;138(6):1075–89. doi: 10.1007/s00401-019-02056-2 (PMC6851394; doi:10.1007/s00401-019-02056-2)
Supplement: Supplementary file 7 — Supplementary material 7 (DOCX 14 kb). Summary of the histological, radiological, and clinical features of each patient [file 401_2019_2056_MOESM7_ESM.docx]

| Pat. Num. | Age at diag. | Gender | Hist.  diag. | Vertebral level | Radiological features of the tumor | Dissemination at diag. | No. of relapses/ progression | Surgery | RTx | CTx & used agents | Targeted Therapy | PFS  (months) | OS  (months) | Status |
| --- | --- | --- | --- | --- | --- | --- | --- | --- | --- | --- | --- | --- | --- | --- |
| 1 | 14 | f | EPN, WHO II | C7 – T2 | Nodular and leptomeningeal enhancement,  perifocal edema, intratumoral cysts | No | 8 | STR (4x) | Yes (5x) | Yes (5x)  VCR/CPH, CP/ETO  TMZ  TFS/EPO | Bevacizumab | 19 | 91 | Dead |
| 2 | 18 | m | Ana. EPN, WHO III | T8 | NA | Yes (spinal) | 5 | STR (3x) | Yes (5x) | Yes (3x)  CP/ETO, CP | Imatinib | 31 | 111 | Alive |
| 3 | 12 | m | Ana. EPN, WHO III | C7-Th6, Th7-8, Th9-10 | NA | Yes (spinal & intracranial) | 5 | STR (1x) | Yes (6x) | Yes (3x)  TMZ  CPH/ETO | Bevacizumab | 12 | 61 | Dead |
| 4 | 35 | f | Ana. EPN, WHO III | C2-T3 | NA | NA | 2 | STR (1x) | Yes (2x) | Yes (1x) | Trametinib  Bevacizumab | 15 | 29 | Alive, pall. care |
| 5 | 34 | m | Ana. EPN, WHO III | L5 | Nodular and leptomeningeal enhancement, intratumoral cysts | Yes (spinal) | 3 | STR (1x) | Yes (3x) | Yes (2x)  TMZ | No | 4 | 19 | Dead |
| 6 | 26 | f | Ana. EPN, WHO III | T10 -12 | Nodular and leptomeningeal enhancement | Yes (spinal) | 3 | STR (3x) | Yes (1x) | Yes (2x)  TMZ  CP/ETO | No | 17 | 31 | Alive |
| 7 | 23 | m | Ana. EPN, WHO III | T6-11 | NA | Yes (spinal & intracranial) | 2 | STR (1x) | Yes (1x) | Yes (3x)  TMZ  CP/ETO | No | 79 | 103 | LoF |
| 8 | 32 | f | EPN, WHO II | T7-9 | Nodular and leptomeningeal enhancement | Yes (spinal) | 1 | STR (2x) | Yes (1x) | Yes (1x)  TMZ | No | 34 | 46 | Dead |
| 9 | 56 | f | Ana. EPN, WHO III | NA | NA | NA | NA | GTR (1x)  Total: 3x | Yes (NA) | No | No | 8 | 87 | Dead |
| 10 | 35 | m | Ana. EPN, WHO III | C6/7, T2/3, C7-T3, T3-5 | Leptomeningeal enhancement | Yes (spinal) | 0 | STR (1x) | Yes (1x) | Yes (1x)  TMZ | No | 4 | 4 | Alive |
| 11 | 37 | m | EPN, WHO II | C5-7, T4-5 | Leptomeningeal enhancement, intratumoral cysts | Yes (spinal) | 0 | STR (1x) | Yes (1x) | No | No | 8 | 8 | Alive |
| 12 | 46 | f | Ana. EPN, WHO III | C7 | Leptomeningeal enhancement, intratumoral cysts | Yes (spinal & intracranial) | 1 | No | No | Yes  CP/ETO | No | 2 | 2 | Alive |
| 13 | 16 | f | Ana. EPN, WHO III | NA | NA | NA | NA | Yes (NA) | NA | NA | NA | NA | NA | Dead |

**Suppl. Table 1** Summary of the histological, radiological, and clinical features of each patient.
